# Assessing the maxillary sinus mucosa of rabbits in the presence of biodegradable implants

**DOI:** 10.5935/1808-8694.20120031

**Published:** 2015-10-20

**Authors:** André Coura Perez, Armando da Silva Cunha, Sílvia Ligório Fialho, Lívia Mara Silva, João Vicente Dorgam, Adriana de Andrade Batista Murashima, Alfredo Ribeiro Silva, Maria Rossato, Wilma Terezinha Anselmo-Lima

**Affiliations:** a MD (PhD student); b PhD (Associate Professor); c PhD (Pharmacotechnical Development Manager); d MD (Undergraduate student); e MD (PhD student); f MD (MSc student); g PhD (Professor at the Ribeirão Preto Medical School at the University of São Paulo); 8 Laboratory Technician (Ribeirão Preto Medical School at the University of São Paulo); 9 Associate Professor (Professor)

**Keywords:** drug implants, histology, prednisolone, sinusitis

## Abstract

In an attempt to improve the quality of life of patients with vitreous humor disease, ophthalmologists began offering steroid-eluting biodegradable implants to their patients. These implants can be used as an alternative treatment for CRS and this is why this experimental study was carried out on rabbit maxillary sinuses.

**Objective:**

This study aims to assess the histology of the mucosa of the maxillary sinuses of rabbits after the placement of a prednisolone-eluting biodegradable implant.

**Method:**

Eighteen rabbits were randomly divided into two groups: group 1 - subjects had drug-eluting implants placed on their left maxillary sinuses; group 2 - subjects had non-drug-eluting implants placed on their left maxillary sinuses. The right maxillary sinuses served as the controls. After seven, 14, and 28 days three rabbits in each group were randomly picked to have their tissue inflammatory response assessed.

**Results:**

Levels of mucosal inflammation were not significantly different between the groups with and without drug-eluting implants and the control group, or when the groups with drug-eluting implants and non-drug-eluting implants were compared.

**Conclusion:**

Signs of toxicity or mucosal inflammation were not observed in the maxillary sinuses of rabbits given prednisolone-eluting implants or non-drug-eluting implants.

## INTRODUCTION

Chronic rhinosinusitis (CRS) is one of the most common health problems to affect the population. Health care costs incurred in to treat this condition are significant. About 135 in every 1,000 people in the United States - or 31 million people - are affected every year at a total cost of 6 billion US dollars^1^, ^2^, ^3^.

The pathophysiology of CRS is uncertain to this date, and the most widely accepted theory around it states that it is a multifactorial chronic inflammatory disease possibly associated with genetic predisposition. Some of the related factors include: biofilm, osteitis, allergy, immune disorders, upper airway intrinsic factors, *Staphylococcus aureus* super antigens, eosinophilic inflammation produced by fungal infection, and metabolic disorders such as hypersensitivity to aspirin^3^. Many inflammatory patterns are believed to be involved with CRS and that they may differ depending on the postoperative prognosis^3^, ^4^.

Various therapies have been suggested for patients with CRS. High grade evidence indicates that topical nasal steroids, systemic steroids, and low-dosage macrolides on long term regimens are effective in managing CRS. Functional endoscopic sinus surgery (FESS) may be an alternative to patients failing to respond to the medical treatment^5^.

The lack of knowledge on the actual pathophysiology of CRS translates into difficulties finding a curative therapy, thus depositing additional importance on patient compliance and treatment preferences. Mode of administration, type of medication, total cost of treatment and side effects must always be considered.

Intraocular injections of steroids are a frequent nuisance in the lives of patients with vitreous humor disease. In order to spare them from the injections, ophthalmologists have been offering steroid-eluting biodegradable implants to their patients^6^, ^7^.

The placement of a biodegradable implant on the target organ allows better control of drug administration and the attainment of therapeutical levels with lower drug concentrations, virtually eliminating adverse systemic effects^8^, ^9^, ^10^. Non-biodegradable implants have also been used to the same end with promising results. Nonetheless, they require a second procedure to remove the implant after all the drug has been released^8^.

Biodegradable implants are prepared from various polymers which, in vivo, must ideally degrade for enough time to allow controlled drug release and thus produce biocompatible metabolites that can be easily washed away^11^.

The most frequently used biodegradable polymers are polyesters such as caprolactone, o polylactic acid (PLA) and various lactic and glycolic acid copolymer types (PLGA), the latter two being extensively used^12^.

**Figure 1:**
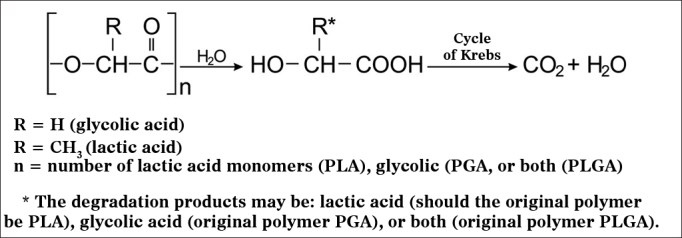
Hydrolysis mechanism of PLA, PGA or PLGA (adapted from Merkli et al.^13^).

PLA and the different types of PLGA have been widely used and studied in prolonged drug delivery systems (PDRS) in various human tissues. Biodegradation of these polymers occurs by erosion, cleavage of the polymer chain by hydrolysis with the consequent release of lactic and glycolic acids. These acids are natural metabolites and are eliminated by the Krebs cycle in the form of carbon dioxide and water (Figure 1)^13^.

The presence of a methyl (CH3) group in the lactic acid chain of PLGA confers greater hydrophobicity to the biomaterial when compared to byproducts containing greater amounts of glycolic acid (PGA). Therefore, PGA is quite sensitive to hydrolysis and unfit to be used in PDRS. As it concerns PLGA, the greater the levels of lactic acid, the more hydrophobic the copolymer, the less water it absorbs, and the slower it degrades. Additionally, molecular weight and degree of crystallinity may impact the mechanical properties, hydrolytic capacity, and degradation rates of these polymers^9^.

PDRS using PLGA with high contents of lactic acid - characterized by lower degradation rates - may result in systems in which drug release occurs for periods of time as long as three years, depending on production parameters and external variables related to the tissue in which it is placed^14^. Thus, the development of these systems may mean significant progress, once they are able to keep drug concentrations on the desired site within therapeutical levels for a long period of time^15^, ^16^, ^17^, ^18^, ^19^. Many drugs may be conveyed using implants, such as antibiotics, antiviral drugs, chemotherapy agents, and steroids, with satisfactory results in eye care according to recently published papers, in which implant degradation in the vitreous did not lead to damage to eye tissues^6^, ^8^, ^13^.

Implants may become a good alternative treatment to CRS, as once in contact with the paranasal sinus mucosa they may facilitate treatment and patient compliance, acting as a substitute for the daily application of topical steroids. Therefore, this study aims to assess the histology of the mucosa of the maxillary sinuses of rabbits after the placement of a prednisolone-eluting biodegradable implant.

## METHOD

This study included 18 female New Zealand rabbits weighing between 2.5 and 3.5 Kg kept in cages in the animal lab of the Ribeirão Preto Medical School of the University of São Paulo (FMRP-USP). The 18 subjects were randomly divided into two groups.

**Figure 2:**
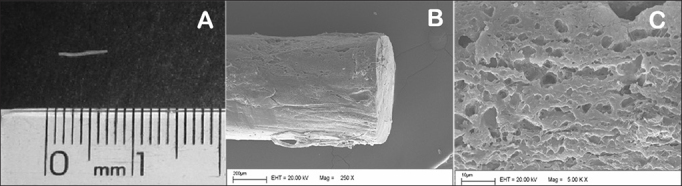
A: Macroscopic view of biodegradable implant; B: SEM image of biodegradable implant; 250x magnifcation; C: SEM images of biodegradable implant; 5000x magnifcation.

In the preparation of the biodegradable implants, prednisolone and PLGA (75:25) were weighed on a ratio of 21-26/79-74% and then solubilized on proper solvent and distilled water. The solution was then filtered using a sterile 0.2 mm filter under laminar flow. Next it was frozen dried, and used to prepare hot-molded rod-shaped implants. The implants weighed between 0.9 and 1.2 mg, measured 4.5 to 6.0 mm in length, and had diameters ranging between 0.4 and 0.5 mm (Figure 2A). The low coefficient of variation between the implants was indicative of the reproducibility of the employed technique. Macroscopically, the implants were similar smooth monolithic systems with steroids scattered within the polymer matrix. The surface and outer shape of the implants were examined on an scanning electron microscope (SEM). Surface images were captured at magnification powers ranging from 250 to 5,000 times (Figure 2B-C).

### Surgical Procedure

**Figure 3:**
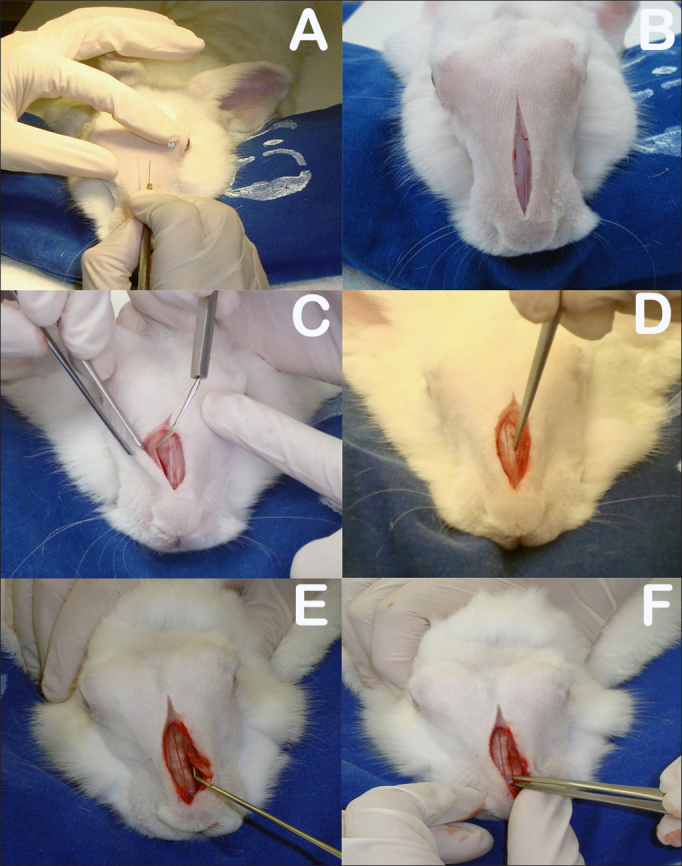
A: Sagittal incision on rabbit dorsum; B: Five-centimeter sagittal incision to periosteum; C: Detachment of periosteum to expose bone suture on the midline and anterior walls of both maxillary sinuses; D: Rectangular bone-mucosal fap produced with chisel and hammer; E: Placement of prednisolone-eluting biodegradable implant in left maxillary sinus; F: Bone fap in place.

The rabbits were anesthetized with intramuscular xylazine hydrochloride (20 mg/Kg) and ketamine hydrochloride (10 mg/Kg). The nasal dorsum of the rabbits was cleaned with iodine. A sagittal incision of approximately 5 cm on the midline of the nasal dorsum down to the periosteum was performed (Figure 3A-B). The periostea on both sides of the nasal dorsum were detached with a Paparella ear tube to expose the bone suture on the midline and the anterior wall of both maxillary sinuses (Figure 3C). Chisel and hammer were used to produce 25x8 mm rectangular bone-mucosal flaps on the anterior wall of both maxillary sinuses, with the larger axis running parallel to the midline bone suture. The medial border of the flap was 3 mm away from the midline bone suture and the upper border 3 to 5 mm below the frontal-maxillary suture (Figure 3D). The macroscopic aspect of the maxillary sinus mucosa was observed with the aid of a headlight.

The rabbits were divided into two groups. Group 1 subjects had prednisolone-eluting biodegradable implants placed on their left maxillary sinuses; the same procedure was carried out in their right maxillary sinuses, but no implants were inserted (Figure 3E); Group 2 had non-drug-eluting biodegradable implants placed on their left maxillary sinuses; the same procedure was carried out in their right maxillary sinuses, but no implants were inserted.

The right maxillary sinuses of both groups were used as controls. After this stage, the bone flaps were put in place again to mitigate invasion on sinus tissue and the subjects' skin was stitched closed with nylon 4-0 wire to ensure tight contact between the periosteum and subcutaneous tissue (Figure 3F). The site of the procedure was again cleaned with iodine and subjects were administered intramuscular ketoprofen (10mg/kg) for two days and enrofloxacin (0,1 ml/kg) for three days.

The experimental protocol employed in this study was approved by the Animal research Ethics Committee at FMRP-USP (permit n^o^ 132/2010).

**Figure 4:**
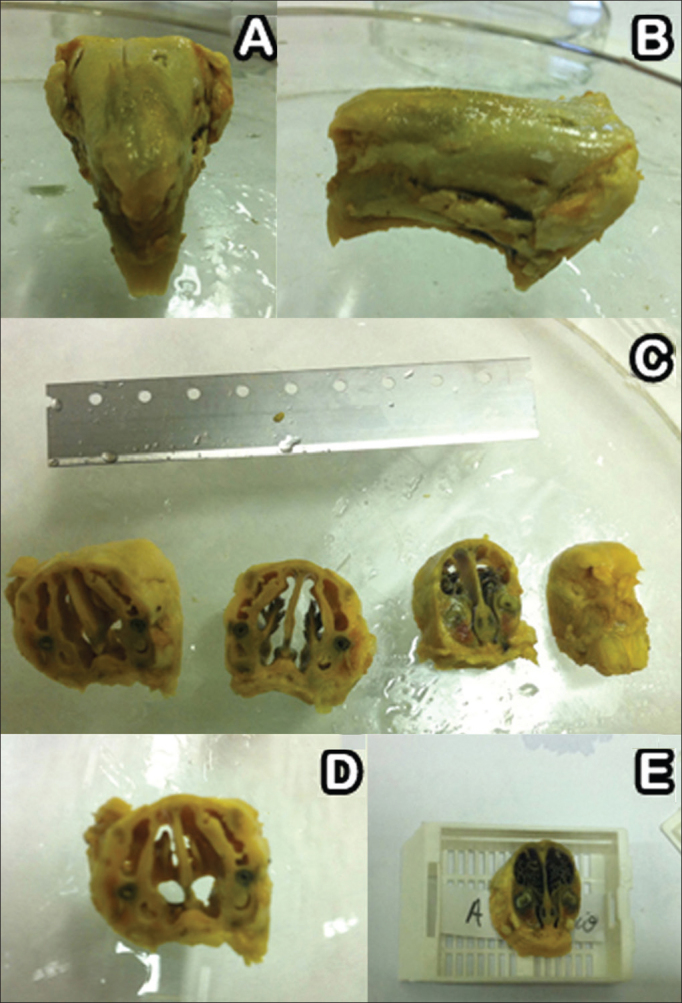
A-B: Dehydrated facial mesostructure in 10% formaldehyde; C-E: Serial cross-sectional slices from nose to skull every two centimeters.

After seven, 14, and 28 days three rabbits were randomly picked from each group and slaughtered under general anesthesia as described above. Their facial mesostructure was removed in a block resection and placed in 10% formaldehyde for further histological analysis (Figure 4A-B). For histology analysis purposes, the specimens were placed in a formaldehyde solution (10% w/v) and were left for six days in a solution of nitric acid (5% w/v) for decalcification. Serial cross-sectional slices from the nose to the skull were made every two centimeters (Figure 4C-E); then they were dehydrated, clarified, and embedded in paraffin. Next, the specimens were cut on a microtome in 5 µm thick slices, and stained in hematoxylin-eosin to assess tissue inflammatory response patterns.

The slides were analyzed by the same pathologist using a semiquantitative approach. The examiner was blinded for the groups to which the slides belonged, and rated them as per the adopted criteria for inflammation: 0 = no inflammation; 1 = mild inflammation (scattered inflammatory cells, no evident epithelial lesion); 2 = moderate inflammation (diffuse inflammatory infiltrate in the lamina propria, no formation of inflammatory aggregates, presence of focal epithelial cell lesion characterized by disorganized ruptured epithelial cells); 3 = severe inflammation (dense diffuse inflammatory infiltrate with formation of inflammatory aggregates and diffuse epithelial cell lesion characterized by disorganized ruptured epithelial cells)^20^.

Software package SPSS (Statistical Package for Social Sciences) release 19.0 was used to process raw data and produce statistical results. A statistical significance level of 5% (0.05) was adopted in statistical tests.

## RESULTS

**Figure 5:**
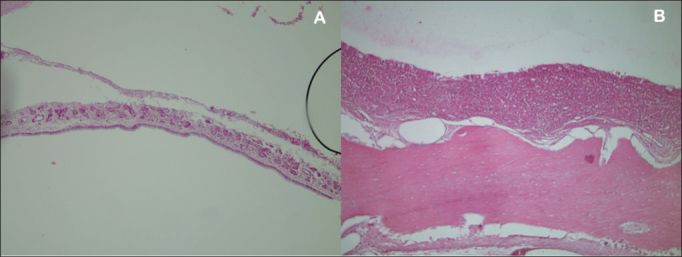
A: Left maxillary sinus mucosa (rabbit 1) without infamma-tion or sings of toxicity (HE stain, 10x magnification); B: Left maxillary sinus mucosa (rabbit 5) with moderate inflammation (HE stain, 10x magnifcation).

Seven rabbits in Group 1 (prednisolone-eluting biodegradable implants on left maxillary sinuses) did not present signs of mucosal inflammation; two were slaughtered on day 7, three on day 14, and two on day 28 after implantation. Only two, one slaughtered on day 7 and another on day 28 after implantation, had mild and moderate inflammation respectively (Figure 5). Severe inflammation was observed in the right maxillary sinuses (control side) of two rabbits slaughtered seven days after implantation, whereas the rest of the group had no signs of inflammation (Table 1). Mild mucosal inflammation was observed in one of the rabbits slaughtered seven days after implantation and in two slaughtered on day 14 in Group 2 (non-drug-eluting biodegradable implants on left maxillary sinuses). Moderate mucosal inflammation was seen in two rabbits slaughtered on days 7 and 28 respectively (Figure 5A-B). Only one subject slaughtered on day 28 had severe inflammation. The remaining subjects did not have signs of inflammation. Four rabbits in Group 2 (controls) did not have right maxillary sinus mucosal inflammation. Mild inflammation was observed in three subjects; moderate inflammation in one and severe in another slaughtered on day 28 (Table 2).

**Table 1:** Histological assessment of inflammation on mucosas of rabbits in Group 1.

Subjects	Right maxillary sinus (control)	Left maxillary sinus	Time to slaughter
C13	0	(I + P) 0	7 days
C17	0	(I + P) 1	7 days
C18	0	(I + P) 0	7 days
C1	0	(I + P) 0	14 days
C2	0	(I + P) 0	14 days
C3	0	(I + P) 0	14 days
C6	3	(I + P) 2	28 days
C7	3	(I + P) 0	28 days
C8	0	(I + P) 0	28 day

0: no inflammation; 1: mild inflammation; 2: moderate inflammation; 3: severe inflammation; (I+P): prednisolone-eluting biodegradable implant.

**Table 2:** Histological assessment of inflammation on mucosas of rabbits in Group 2.

Subjects	Right maxillary sinus (control)	Left maxillary sinus	Time to slaughter
C14	0	(I) 1	7 days
C15	1	(I) 0	7 days
C16	0	(I) 2	7 days
C10	0	(I) 0	14 days
C11	1	(I) 1	14 days
C12	1	(I) 1	14 days
C4	0	(I) 0	28 days
C5	2	(I) 2	28 days
C9	3	(I) 3	28 days

0: no inflammation; 1: mild inflammation; 2: moderate inflammation; 3: severe inflammation; (I): biodegradable implant without prednisolone.

### Statistical Results

#### Description and comparison between sample members

McNemar's test was applied to verify possible differences between both investigated sides for variable ‘inflammation' in two categories (Table 3). No statistically significant differences were noted on inflammatory patterns when the mucosas of the maxillary sinuses receiving the implants with and without prednisolone were compared to their controls.

**Table 3:** Inflammation pattern in controls and sinuses with biodegradable implants.

Controls	Sinuses with biodegradable implants		Total
	N	Y	
N	8	3	11
	44.40%	16.70%	61.10%
Y	2	5	7
	11.10%	27.80%	38.90%
Total	10	8	18
	55.60%	44.40%	100.00%

*p* > 0.999.

#### Description and comparison between implants with and without steroids

Fisher's exact test was used to verify possible differences between implants with and without steroids for variable ‘inflammation' in two categories (Table 4). A trend indicative of lesser mucosal inflammation in the rabbits given the implant with steroids was found when the mucosas of the maxillary sinuses of rabbits receiving the implants with prednisolone were compared to the mucosas of the subjects receiving the implants without prednisolone, as the calculated p-value sat between 5% (0.050) and 10% (0.100).

**Table 4:** Inflammation pattern in maxillary sinuses with and without prednisolone.

Implants	Sinuses with biodegradable implants		Total
	N	Y	
P	7	2	9
	77.80%	22.20%	100.00%
Non-P	3	6	9
	33.30%	66.70%	100.00%
Total	10	8	18
	55.60%	44.40%	100.00%

*p* = 0.077.

#### Effect of variable ‘slaughter time'

A verisimilitude ratio test was applied to verify possible differences between the three times at which the rabbits were slaughtered when compared concurrently for the variables of interest (Tables 5 and 6). No statistically significant differences were found in mucosal inflammation when the groups receiving implants with and without steroids were compared to controls, or when the group receiving the implants with prednisolone was compared to the group receiving the implants without steroids.

**Table 5:** Inflammation pattern in control sinuses vs. time to slaughter.

Time to slaughter	Control Sinus		Total
	No	Yes	
7 days	5	1	6
	83.30%	16.70%	100.00%
14 days	4	2	6
	66.70%	33.30%	100.00%
28 days	2	4	6
	33.30%	66.70%	100.00%
Total	11	7	18
	61.10%	38.90%	100.00%

*p* = 0.185.

**Table 6:** Inflammation pattern in sinuses with biodegradable implants vs. time to slaughter.

Time to slaughter	Sinus with biodegradable implant		Total
	No	Yes	
7 days	3	3	6
	50.00%	50.00%2	100.00%
14 days	4		6
	66.70%	33.30%	100.00%
28 days	3	3	6
	50.00%	50.00%	100.00%
Total	10	8	18
	55.60%	44.40%	100.00%

*p* = 0.796; P: with prednisolone; non-P: no prednisolone; Y: (yes) inflammation present; N: (no) inflammation absent.

## DISCUSSION

The exact pathogenesis of CRS is yet unknown. Numerous conservative and surgical treatments have been tried without, in many cases, successfully curing patients with the disease. Given that this is a chronic ailment, in which patient compliance to the treatment is essential, the authors of this study decided to try a new mode of delivering the drug to the patients' diseased paranasal sinus mucosas, so that they would no longer have to take daily topical or systemic medication and improve treatment efficacy. Biodegradable implants could be an excellent option: patients would have implants inserted in the mucosa of their noses or paranasal sinuses that carried the proper drug at the ideal therapeutical concentration, without the need to remove them later, as they would be absorbed by the patients' bodies.

On a first study done in 2006^7^ and in another two carried out in 2007 and 2008, Fialho et al.^8^, ^10^ showed that steroid-eluting biodegradable implants placed on the vitreous of rabbits' eyes did not lead to toxicity or significant inflammatory response, as similarly seen in the mucosas of the paranasal sinuses of the rabbits included in this study. However, it is worthy noting that in the ENT area this is the first study to describe the use of biodegradable implants as a means to provide controlled release of medication (prednisolone) onto paranasal sinuses.

This study was limited to assessing the inflammation these implants could introduce to the mucosas of the maxillary sinuses of rabbits. Other parameters need to be further studied, such as the proper concentration of the drug on the implant and the ideal amount of polymer to allow for optimal implant degradation and, consequently, the amount of medication to be released onto the target organ, the best implant size to reach each specific goal, and how much drug could be absorbed systemically. This is key information for the future development of implants that can be safely placed in humans as seen in ophthalmic care.

This study has been the first step toward the development of a possible treatment for CRS.

## CONCLUSION

Although this is a preliminary study, we may state that the implants with and without steroids did not cause toxicity or inflammation to the maxillary sinus mucosas of rabbits.
